# Inferring and analyzing gene regulatory networks from multi-factorial expression data: a complete and interactive suite

**DOI:** 10.1186/s12864-021-07659-2

**Published:** 2021-05-26

**Authors:** Océane Cassan, Sophie Lèbre, Antoine Martin

**Affiliations:** 1 BPMP, CNRS, INRAE, Institut Agro, Univ Montpellier, Montpellier, 34060 France; 2 IMAG, Univ. Montpellier, CNRS, Montpellier, France; 3 Université Paul-Valéry-Montpellier 3, Montpellier, France

**Keywords:** Gene regulatory network inference, Graphical user interface, Multifactorial transcriptomic analysis, Model-based clustering, Analysis workflow

## Abstract

**Background:**

High-throughput transcriptomic datasets are often examined to discover new actors and regulators of a biological response. To this end, graphical interfaces have been developed and allow a broad range of users to conduct standard analyses from RNA-seq data, even with little programming experience. Although existing solutions usually provide adequate procedures for normalization, exploration or differential expression, more advanced features, such as gene clustering or regulatory network inference, often miss or do not reflect current state of the art methodologies.

**Results:**

We developed here a user interface called DIANE (Dashboard for the Inference and Analysis of Networks from Expression data) designed to harness the potential of multi-factorial expression datasets from any organisms through a precise set of methods. DIANE interactive workflow provides normalization, dimensionality reduction, differential expression and ontology enrichment. Gene clustering can be performed and explored via configurable Mixture Models, and Random Forests are used to infer gene regulatory networks. DIANE also includes a novel procedure to assess the statistical significance of regulator-target influence measures based on permutations for Random Forest importance metrics. All along the pipeline, session reports and results can be downloaded to ensure clear and reproducible analyses.

**Conclusions:**

We demonstrate the value and the benefits of DIANE using a recently published data set describing the transcriptional response of Arabidopsis thaliana under the combination of temperature, drought and salinity perturbations. We show that DIANE can intuitively carry out informative exploration and statistical procedures with RNA-Seq data, perform model based gene expression profiles clustering and go further into gene network reconstruction, providing relevant candidate genes or signalling pathways to explore. DIANE is available as a web service (https://diane.bpmp.inrae.fr), or can be installed and locally launched as a complete R package.

**Supplementary Information:**

The online version contains supplementary material available at (10.1186/s12864-021-07659-2).

## Background

### Analyzing gene expression to uncover regulatory mechanisms

A multitude of regulatory pathways have evolved in living organisms in order to properly orchestrate development, or to adapt to environmental constraints. Much of these regulatory pathways involve a reprogramming of genome expression, which is essential to acquire a cell identity corresponding to given internal and external environments. To characterize these regulatory pathways, and translate these changes in gene expression at the genome-wide level, global transcriptome study under various species, tissues, cells and biological conditions has become a fundamental and routinely performed experiment for biologists. To do so, sequencing of RNA (RNA-Seq) is now the most popular and exploited technique in next-generation sequencing (NGS) methods, and underwent a great expansion in the field functional genomics. RNA-seq will generate fragments, or short reads, that match to genes and quantitatively translate their level of expression. Standard analysis pipelines and consensus methodological frameworks have been established for RNA-Seq. Following quality control of data, reads mapping to a reference genome, and quantification on features of interest are performed, several major steps are commonly found in RNA-Seq data analysis. They usually consist in proper sample-wise normalization, identification of differential gene expression, ontology enrichment among sets of genes, clustering, co-expression studies or regulatory pathways reconstruction.

However, these analysis procedures often require important prior knowledge and skills in statistics and computer programming. In addition, tools dedicated to analysis, exploration, visualization and valorization of RNA-Seq data are very often dispersed. Most of RNA-Seq data are therefore not properly analyzed and exploited at their highest potential, due to this lack of dedicated tools that could be handled and used by (almost) anyone.

### Current tools for facilitating the exploitation of RNA-seq data

Over the last few years, several tools have emerged to ease the processing of RNA-Seq data analysis, by bringing graphical interfaces to users with little programming experience. Among those tools are DEBrowser [1], DEApp [2], iGEAk [3], DEIVA [4], Shiny-Seq [5], IRIS-DEA [6], iDEP [7], or TCC-GUI [8]. All of them propose normalization and low count genes removal, exploratory transcriptome visualizations such as Principal Component Analysis (PCA), and per-sample count distributions plots. They also provide functions for interactive Differential Expression Analysis (DEA) and corresponding visualizations such as the MA-plot. Gene Ontology (GO) enrichment analysis can be performed in those applications, apart from IRIS-DEA, DEApp, and TCC-GUI.

However, when it comes to further advanced analyses such as gene expression profiles clustering or network reconstruction, solutions in those tools are either absent, or sub-optimal in terms of statistical framework or adequacy with certain biological questions. For instance, most of those applications perform clustering using similarity based methods such as k-means and hierarchical clustering, requiring both the choice of metric and criterion to be user-optimized, as well as the selection of the number of clusters. Probabilistic models such as Mixture Models are a great alternative [9–11], especially thanks to their rigorous framework to determine the number of clusters, but they are not represented in currently available tools.

**Fig. 1:**
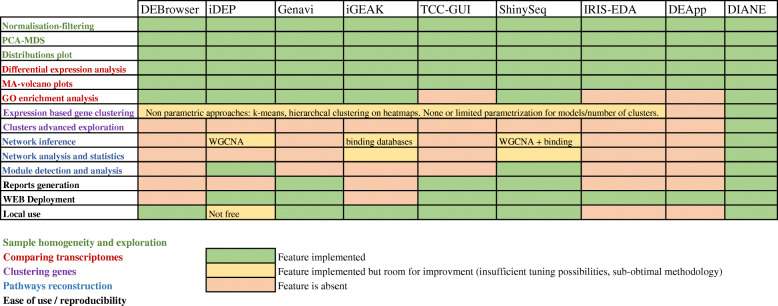
Comparison of tools for facilitating the valorization of expression datasets. Eight interactive tools for analysis of count data from RNA-Seq are presented here and compared in terms of features and methodological choices. The features included are the ones we believe are the expectation from most users willing to exploit RNA-Seq experiments and understand regulatory mechanisms, and that we included to DIANE. Although not reported here for clarity reasons, many compared tools had their own features and specificities of interest. For instance, IRIS-DEA handles single cell RNA-Seq and facilitates GEO submission of the data, iDEP enables to build protein-protein interaction network and has an impressive organisms database, while Shiny-Seq can summarise results directly into power point presentations

Regarding Gene Regulatory Networks (GRN) inference, only three of the applications cited above propose a solution. Two of them, iDEP and Shiny-Seq rely on the popular WGCNA framework (WeiGhted Correlation Network Analysis) [12], which falls into the category of correlation networks. This inference method have the disadvantage of being very vulnerable to false positives as it easily captures indirect or spurious interactions. When the number of samples in the experiment is low or moderate, high correlations are often accidentally found [13]. Besides, linear correlations like Pearson coefficient can miss complex non-linear effects. Lastly, WGCNA addresses the question of co-expression networks, more than GRN. To infer GRN, which should link Transcription Factors (TF) to target genes, iGEAK retrieves information from external interaction databases and binding motives. This allows to exploit valuable information, but makes this step extremely dependent on already publicly available datasets. An exhaustive comparison with respect to the features and methods handled by the described interfaces for RNA-Seq analysis is given in Fig. 1.

Other frameworks focus on gene network reconstruction and visualization only. For instance, the web server GeNeCK [14] makes the combination of several probabilistic inference strategies easily available, but there is no possibility to select a subset of genes to be considered as regulators during inference. The online tool ShinyBN [15] performs Bayesian network inference and visualization. This Bayesian approach is however prohibitive when large scale datasets are involved. Lastly, neither ShinyBN nor GeNecK allow for upstream analyses and exploration of RNA-Seq expression data.

Consequently, efficient statistical and machine learning approaches for GRN inference (like for instance GENIE3 [16], TIGRESS [17], or PLNModels [18], see [19] for a review) are not available, to our knowledge, as a graphical user interfaces allowing necessary upstrem operations like normalization or DEA.

Besides, all of the cited applications are available as online tools or as local packages with source code, although the useful possibility to provide both solutions simultaneously, in order to satisfy advanced users as much as occasional ones, is not always available. It is also worth noting that availability of organisms in current services varies a lot. Some of them like iGEAK are restricted to human or mouse only.

### Proposed approach

In this article, we propose a new R-Shiny tool called DIANE (Dashboard for the Inference and Analysis of Networks from Expression data), both as an online application and as a fully encoded R package. DIANE performs gold-standard interactive operations on RNA-Seq datasets, possibly multi-factorial, for any organism (normalization, DEA, visualization, GO enrichment, data exploration, etc.), while pushing further the clustering and network inference possibilities for the community. Clustering exploits Mixture Models including RNA-seq data prior transformations [11] and GRN inference uses Random Forests [16, 20], a non-parametric machine learning method based on a collection of regression trees. In addition, a dedicated statistical approach, based on both the biological networks sparsity and the estimation of empirical *p*-values, is proposed for the selection of the edges. Step-by-step reporting is included all along the analyses, allowing reproducible and traceable experiments.

In order to illustrate the different features of DIANE, we have used a recently published RNA-seq data set, describing the combinatorial effects of salt (S), osmotic (M), and heat (H) stresses in the model plant *Arabidopsis thaliana* [21]. RNA-seq were performed under single (H, S, M), double (SM, SH, MH), and triple (SMH) combinations of salt, osmotic, and heat stresses. In the course of our paper, we will demonstrate that DIANE can be a simple and straightforward tool to override common tools for transcriptome analyses, and can easily and robustly lead to GRN inference and to the identification of candidate genes.

## Implementation and results

**Fig. 2:**
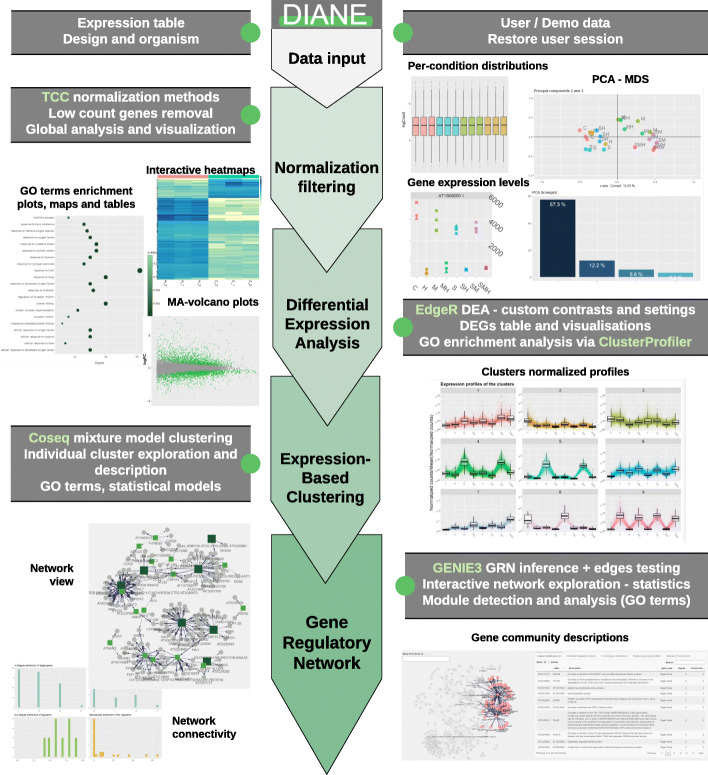
DIANE’s workflow. The main steps of the pipeline available in the application -data import, normalization, exploration, differential expression analyses, clustering, network inference- alongside with some chosen visual outputs

DIANE is an R Shiny [22, 23] application available as an online web service, as well as a package for local use. To perform relevant bioinformatic and bio-statistical work, different existing CRAN and Bioconductor packages as well as novel functions are brought together. Its development was carried out via the golem [24] framework, allowing a modular and robust package-driven design for complex production-grade Shiny applications. Each main feature or analysis step is programmed as a shiny module, making use of the appropriate server-side functions. In the case of local use, those functions are exported by the package so they can be called from any R script to be part of an automated pipeline or more user-specific analyses. We also provide a Dockerfile [25] and instructions so that interested users can deploy DIANE to their own team servers. Figure 2 presents the application workflow and main possibilities. The analysis steps in DIANE are shown in a sequential order, from data import, pre-preprocessing and exploration, to more advanced studies such as co-expression or GRN inference.

### Data upload

#### Expression file and design

To benefit from the vast majority of DIANE’s features, the only required input is an expression matrix, giving the raw expression levels of genes for each biological replicate across experimental samples. It is assumed that this expression matrix file originates from a standard bioinformatics pipeline applied to the raw RNA-Seq fastq files. This typically consists in quality control followed by reads mapping to the reference genome, and quantification of the aligned reads on loci of interest.

#### Organism and gene annotation

Several model organisms are included in DIANE to allow for a fast and effortless annotation and pathway analysis. For now, automatically recognized model organisms are *Arabidopsis thaliana*, *Homo sapiens*, *Mus musculus*, *Drosophilia melanogaster*, *Ceanorhabditis elegans*, and *Escherichia coli*. DIANE takes advantage of the unified annotation data for those organisms offered by the corresponding Bioconductor organisms database packages [26–31]. Other plant species are annotated such as white lupin, and users can easily upload their custom files to describe any other organism whenever it is needed or possible along the pipeline. Organism specific information needed can be common gene names and descriptions, gene - GO terms associations, or known transcriptional regulators.

### Normalization and low count genes removal

DIANE proposes several strategies of normalization to account for uneven sequencing depth between samples. One step normalization can be performed using either the Trimmed Mean of M values method (TMM) [32] or the median of ratios strategy from DESeq2 [33]. The TCC package [34] also allows to perform a prior DEA to remove potential differentially expressed genes (DEG), and then compute less biased normalization factors using one of the previous methods. DIANE also includes a user-defined threshold for low-abundance genes, which may reduce the sensitivity of DEG detection in subsequent analyses [35]. The effect of normalization and filtering threshold on the count distributions can be interactively observed and adjusted.

### Exploratory analysis of RNA-seq data

#### PCA - MDS

Dimensionality reduction techniques are frequently employed on normalized expression data to explore how experimental factors drive gene expression, and to estimate replicate homogeneity. In particular, the Multi-Dimensional Scaling (MDS) plot takes samples in a high dimensional space, and represents them as close in a two-dimensional projection plane [36] depending on their similarity. Principal Component Analysis (PCA) is also a powerful examination of expression data. Through linear algebra, new variables are built as a linear combination of the initial samples, that condense and summarize gene expression variation. By studying the contribution of the samples to each of these new variables, the experimenter can assess the impact of the experimental conditions on gene expression. DIANE offers those two features on expression data, where each gene is divided by its mean expression to remove the bias of baseline expression intensity.

**Fig. 3:**
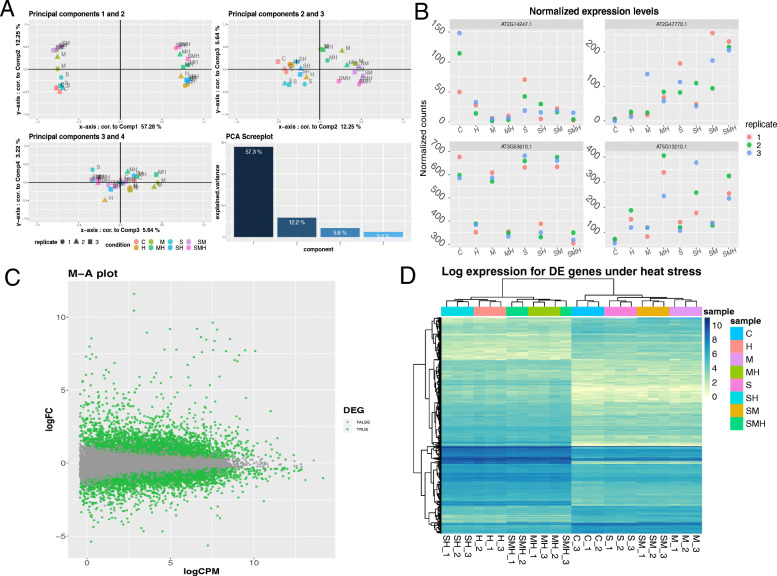
Normalization and exploration of RNA-seq dataset with DIANE. **a** PCA analysis for the normalized expression table. The experimental conditions have for coordinates their contributions (correlations) to the first four principal components. The scree-plot shows, for each principal component, the part of global variability explained. **b** Example of normalized gene expression levels across all seven perturbations and control. **c** MA-plot for the DEG in response to a single heat stress. The x-axis is the average expression, and the y-axis is the LFC in expression between heat stress and control. DEG with FDR <0.05 and an absolute LFC >2 appear in green. **d** Log normalized expression heatmap for the DEG under heat across all perturbations and control

As presented in Fig. 3a, we applied PCA to the normalized transcriptomes after low gene counts removal. No normalization was applied in DIANE as raw data was presented as Tags Per Millions. We found consistent conclusions regarding how heat, salinity and osmotic stresses affect gene expression. The first principal component, clearly linked to high temperature, discriminates the experimental conditions based on heat stress while explaining 57% of the total gene expression variability. The second principal component, to which mannitol-perturbed conditions strongly contributes, accounts for 12% of gene expression variability. The effect of salinity is more subtle and can be discerned in the third principal component.

#### Normalized gene expression profiles

The "expression levels" tab of the application is a simple exploratory visualization, that allows the user to observe the normalized expression levels of a several genes of interest, among the experimental conditions of its choice. Each replicate is marked as different shapes. Besides rapidly showing the behavior a desired gene, it can provide valuable insights about a replicate being notably different from the others.

Using this feature of DIANE, we represented in Fig. 3b four genes showing different behaviors in response to the combination of stresses, and illustrating the variation that can be found among biological replicates.

### Differential expression analysis

DEA in DIANE is carried out through the EdgeR framework [37], which relies on Negative Binomial Modelling. After gene dispersions are estimated, Generalized Linear Models are fitted to explain the log average gene expressions as a linear combination of experimental conditions. The user can then set the desired contrasts to perform statistical tests comparing experimental conditions. The adjusted *p*-value (FDR) threshold and the minimal absolute Log Fold Change (LFC) can both be adjusted on the fly. A data table of DEG and their description is generated, along with descriptive graphics such as MA-plot, volcano plot, and interactive heat-map. The result DEG are stored to be used as input genes for downstream studies, such as GO enrichment analysis, clustering or GRN inference.

Figure 3c and d represent DEG under heat perturbation. Selection criteria were adjusted *p*-values greater than 0.05, and an absolute log-fold-change over 2. The 561 up-regulated genes and 175 down-regulated genes are indicated in green in the MA-plot, and correspond to the rows of the heatmap. The high values of LFC for those genes, along with their expression pattern in the heatmap across all conditions confirm the strong impact of heat stress on the plants transcriptome.

In the case where several DEA were performed, it might be useful to compare the resulting lists of DEG. DIANE can perform gene lists intersection, and provide visualizations through Venn diagrams, as well as the possibility to download the list of the intersection. This feature is available for all genes, or specifically for up or down regulated genes.

### GO enrichment analysis

Among a list of DEG, it is of great interest to look for enriched biological processes, molecular functions, of cellular components. This functionality is brought to DIANE by the clusterProfiler R package [38], that employs Fischer-exact tests on hypergeometric distribution to determine which GO terms are significantly more represented. Results can be obtained as a downloadable data table, a dotplot of enriched GO terms with associated gene counts and *p*-values, or as en enrichment map linking co-occurring GO terms.

### Gene clustering

#### Method

In order to identify co-expressed genes among a list of DEGs, DIANE enables gene expression profiles clustering using the statistical framework for inferring mixture models through an Expectation-Maximisation (EM) algorithm introduced by [9, 10]. We chose to use the approach implemented in the Bioconductor Coseq package [11]. Coseq makes it possible to apply transformation to expression values prior to fitting either Gaussian or Poisson multivariate distributions to gene clusters. A penalized model selection criterion is then used to determine the best number of clusters in the data. With DIANE, users simply have to select which DEG should be clustered among previously realized DEA, the experimental conditions to use for clustering, as well as the range of number of clusters to test.

#### Exploring the clusters

Once clustering was performed, a new tab enables a detailed exploration of the created clusters. It includes interactive profiles visualization, downloadable gene data table, GO enrichment analysis. In addition, if the experimental design file was uploaded, Poisson generalized linear models are fitted to the chosen cluster in order to characterize the effect of each factor on gene expression.

To validate and extend the work done around our demonstration dataset, we performed clustering analysis similarly to what was done in the original paper [21]. We considered all genes from the seven DEA computed between control and perturbation treatments, with a 0.05 FDR threshold and an absolute LFC above 2.

**Fig. 4:**
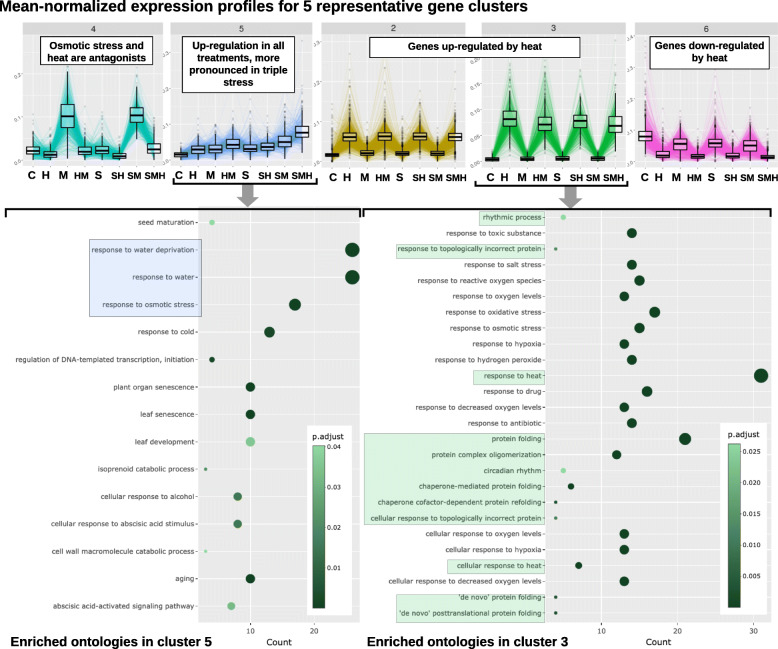
Clustering of combinatorial RNA-seq data with DIANE. Clusters of interest generated by Coseq in DIANE. Gene expression profiles are defined as the normalized expression divided by the mean normalized expression across all conditions. Graphical results of ontology enrichment analysis are presented for clusters 3 and 5. Highlighted ontologies are relevant categories in line with previously published findings [21]. Ontology enrichment plots show detected GO terms (under 0.05 in Fischer’s exact tests), color-coded by their adjusted *p*-value, and shifted in the x-axis depending on the number of genes matching this ontology

Figure 4 presents the clusters of interest as given by the Poisson Mixtures estimation. They provide a gene partitioning representative of all behaviors in the dataset. In particular, we found that the 3 biggest clusters (2, 3, 6) were composed of heat responsive genes. Among those clusters, statistically enriched GO terms are in majority linked to heat and protein conformation. Indeed, proteins misfolding and degradation are direct consequences of high temperatures, thus requiring rapid expression reprogramming to ensure viable protein folding in topology control [39]. Two enriched ontologies involved in rhythmic and circadian processes also support evidence for disrupted biological clock. Second, the cluster 5 brings together genes up-regulated in all stress treatments, with the highest induction being observed in the combination of the three perturbations. Those genes, also noted in [21] to exhibit a synergistic response to mannitol and salt, contain three ontologies related to osmotic stress and water deprivation. Lastly, cluster 4 corroborates the existence of genes characterized by opposite reactions to osmotic stress and heat. They are specifically induced in all mannitol perturbations, except under high temperature, where they are strongly repressed.

### Gene regulatory network inference

GRN inference is a major contribution of DIANE compared to similar existing applications, the latter offering either no possibility for such task, or either limited ones, as described in the “Background” section.

#### Estimating regulatory weights

GRN inference aims to abstract transcriptional dependencies between genes based on the observation of their resulting expression patterns. Each gene is represented by a node in the network. The aim is to recover a weight associated with each edge (i.e. pair of nodes). This is a complex retro-engineering process, challenged by the Curse of dimensionality. Many methods are available, and can be divided into two main categories : statistical and data-driven approaches [13]. Statistical strategies rely on assumptions regarding the data distribution, whose parameters are estimated by maximum-likelihood techniques, often in the case of Bayesian [40] or Lasso inference [17, 41]. However, the underlying modelling assumptions may be inaccurate or difficult to verify in practice. In the second category, the objective is to quantify interaction strengths between pairs of nodes directly from the data. This is typically achieved by using similarity measures such as correlation [12], information theory metrics [42, 43], or feature importances extracted from regression contexts [16]. This second category is less restrictive in terms of hypothesis. However, once the inference is performed, the problem of defining a threshold above which an interaction will be part of the network is far from easy.

There is a large variety of tools available for the task of network inference. Many of them have been benchmarked against one another at the occasion of the DREAM challenges [44, 45]. Those challenges aim at comparing state of the art network inference methods on both simulated and validated biological data. They provide performance metrics for 27 methods based on regression techniques, mutual information metrics, correlation or Bayesian framework among other methods. The performance metrics gathered by DREAM5 [45] (i.e Area Under Precision and Recall curves or overall scores), as well as more recent efforts to compare new methods on those gold standards (i.e F-meausres, ROC curves) are useful resources to help making a choice. For example, existing methods to learn GRN structures are WGCNA [12], ARACNE, CLR, TIGRESS, GENIE3 (see [45] for an exhaustive and referenced list of methods), or also SORDER [46] or CMI2NI [47].

In DIANE, the package chosen for GRN reconstruction is GENIE3 [16], a machine learning procedure that was among the best performers of the DREAM challenges. GENIE3 uses Random Forests [20] which is a machine learning method based on the inference of a collection of regression trees. It has the advantage of being a non-parametric procedure, requiring very few modelling or biological priors, while being able to capture interactions and high order combinatorics between regulators. After having defined a set of regulators among the genes under study, the regression framework allows to infer oriented edges from regulators to targets. With GENIE3, for each target gene, a Random Forest determines the predictive power of each regulator on the target gene expression. The regulatory interactions can then be thresholded according to their importance, so that the strongest links are kept to build a sparse final network. However, choosing such a threshold is not trivial, left as an open question by GENIE3’s authors and ever since.

#### Selecting meaningful regulatory weights

**Fig. 5:**
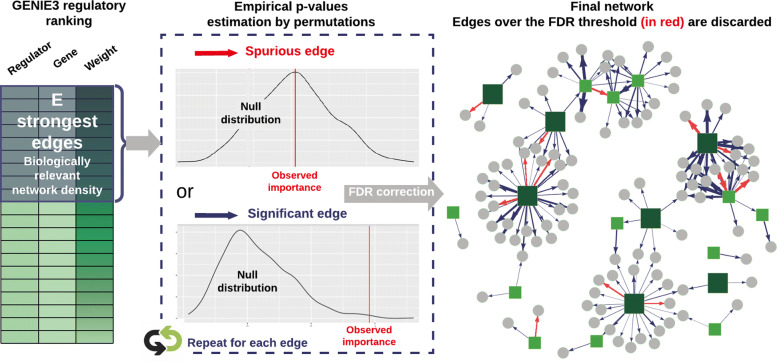
Statistical testing procedure for edges selection among weighted regulatory links. A first network is built from the influence measures resulting from network inference, by choosing an appropriate network connectivity density. The statistical significance of all its edges is then assessed by empirical tests based on permutation for Random Forest importance metrics

**Proposed approach** To avoid the unsatisfying hard-thresholding solution, some researchers make use of TF binding experiments, TF-perturbation assays, or literature data to select a threshold influence measure maximizing prediction precision [48–50]. Network backboning [51, 52] and BRANE Cut [53] are mathematical frameworks that try to extract an informative structure from weighted fully connected networks, but they rely on mathematical modelling and assumptions that we suppose might be too strong or not valid in the precise case of gene regulatory network topology. Feeling the lack of an appropriate model-agnostic strategy with no need for external data, we conceived a method that provides a statistical testing framework for weighted regulator-gene pairs. The main steps of the method, as schematized in Fig. 5, are:

**Inference of the importance values for all regulator-target gene pairs using Random Forests according to GENIE3’s strategy [**16**]** on a chosen list of DEG as input. Transcriptional regulators with a very high value of non linear correlation (typically 0.9 or 0.95) can lead to spurious or missed connections in the final network, and cause robustness issues during the regression procedure. DIANE allows to group them together and to consider them as unique genes.

**Selection of the strongest inferred regulatory influences**. As biological networks are known for their pronounced sparsity [54**–**56], testing all possible regulator-target pairs would be of very little interest, as well as a waste of computation time. We therefore create a first graph, topologically consistent with biological network density standards, which will be further refined by statistical tests.

**Empirical*p*-values are computed for the selected regulatory weights**. To assess weather the importance value of a pair is significant or not, the rfPermute package [57] fits Random Forests and repeatedly shuffles the target gene expression profile so that the null distribution of each regulator influence is estimated. Hence, the empirical *p*-value of a regulator-gene pair is given by the extremeness of its importance as compared to the estimated null distribution. For a faster and more exploratory-oriented network inference, it is possible to skip edges testing (this step and the following).

**FDR correction for multiple testing [**58] is applied to the *p*-values, and only the edges above an FDR threshold are kept to form the final network. After edges statistical testing, graphics that show the *p*-values distribution and the final number of edges depending on the FDR choice are displayed, providing the user with additional decision guidance.

See Additional file 1 for more details on the statistical procedure and implementation. Thanks to this procedure, the main user-defined parameters are the network density prior to statistical tests, and the FDR cut-off. Together, they bring much more biological meaning and decision help than an arbitrary importance threshold.

**Benchmark of the proposed approach** We benchmark this novel procedure designed to keep the most significant interactions from a complete GRN. As GENIE3’s performance was already assessed in several comparative studies, we focus here only on the edges testing strategy, that we compare to a more naive approach, hard thresholding. To do so, we applied our edges selection strategy to GENIE3 edges ranking on two different datasets, for which robust regulator-gene validation information is available.

The first expression dataset is the RNA-Seq experiment on *Arabidopsis thaliana* we present in this article. We inferred a GRN of heat responsive genes in all experimental conditions (1497 genes from C versus H DEA, LFC ≥1.5, FDR ≤0.05, containing 118 regulators). To validate the inferred connections, we made use of connecTF [59], a recent database containing regulatory interactions in Arabidopsis thaliana obtained from in vitro and in vivo binding experiments, as well as in planta regulation experiments. We specifically chose to use the interactions in connecTF obtained from CHIP-Seq and TARGET experiments that represent the most robust data in order to validate connections.

The second dataset is an experiment on *Escherichia coli*, generated by the authors of the "Large-Scale Mapping and Validation of *Escherichia coli* Transcriptional Regulation from a Compendium of Expression Profiles" [60]. We restricted ourselves to a subset of this compendium of experimental conditions corresponding to a single combinatorial experiment. In the latter, bacteria were exposed to a control treatment or to norfloaxacin for different amounts of time, for a total of 24 experimental conditions. The 4345 genes of the organism provided in the dataset, containing 154 transcription factors, are used for GRN inference followed by edges testing. In order to validate the connections of the networks generated in DIANE, we used RegulonDB [61], a database of regulatory interactions built from classic molecular biology experiments and more recently high throughput genomics such as CHIP-Seq and gSELEX.

For each organism, we compared the validity of network predictions between two strategies. The first one corresponds to a network obtained by applying a hard threshold to GENIE3’s weighted regulatory associations, to achieve a desired network connectivity density. The second strategy corresponds to that same network, but after removing the edges deemed spurious by our empirical testing procedure for edges selection. By doing so, we aim at determining weather refining edges with our testing procedure leads to networks of higher quality.

The performance metric we chose to assess our method’s performance is the precision. It is computed as the fraction of edges in the final network that are present in the set of validated interactions, among those for which the regulator possesses validation information in the gold standard (for example, not all regulators were studied in CHIP-Seq nor TARGET experiments, thus are not present in the validated pairs from connecTF).

To provide some parameter exploration, we compare the two strategies for two different initial connectivity densities, and three FDR thresholds to remove spurious interactions. For all the following benchmarks, we used Random Forests made of 1000 trees, and grouped regulators correlated over 90%, as discussed in the previous paragraph "Proposed approach". In order to evaluate robustness while giving an overview of the variability inherent to Random Forest inference and statistical testing by permutations, we launched the two strategies 20 times for each set of parameters and performed non parametric tests for group mean comparisons.

**Fig. 6:**
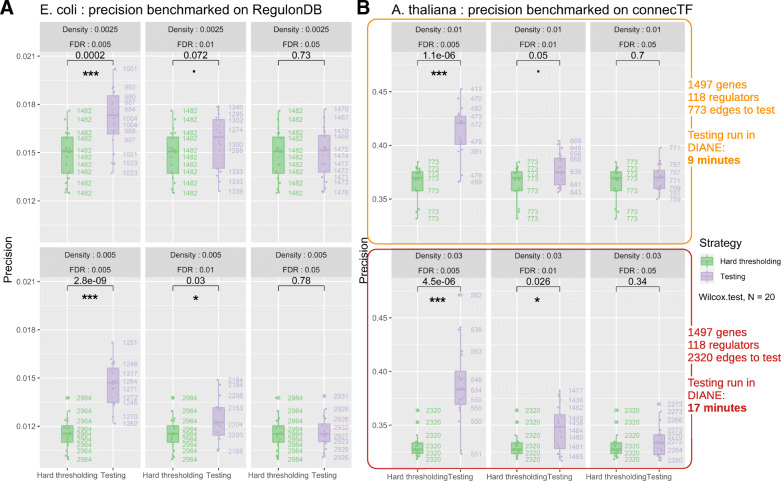
Benchmark of the proposed testing method on the *E. coli* and *A. thaliana* datasets. Boxplots compare the distributions of precision between hard-thresholding (green) and hard-thresholding followed by the removal of non significant edges as predicted by the testing procedure (purple). The 20 replicates for each configuration provide an estimation of the precision dispersion caused by randomness in GENIE3 and testing by permutations. For each organism, we investigate two appropriate connectivity densities, and three adjusted *p*-value thresholds (FDR). On the right of the boxplots, the number of edges kept in the final network are displayed. *P*-values significance of non parametric mean comparisons between the strategies are encoded as follows : 0≤*** <0.001≤** <0.01≤* <0.05≤. <0.1. The results demonstrate that the proposed testing strategy offers a robust gain in precision when using a stringent adjusted *p*-value threshold for edges removal. **a** Results for the GRN inferred on *E. coli* genes, validated on the regulonDB database. **b** Results for the GRN inferred on *A. thaliana* heat-responsive genes, validated on the connecTF database. Additional metrics about the number of genes, interactions to test, and computation time on DIANE’s interface are shown

The results are gathered in Fig. 6a and b. They demonstrate that a significant increase of precision can be achieved on both datasets when choosing stringent adjusted *p*-values for edges removal, independently of prior density. This finding supports that *p*-values obtained from permutations on Random Forest importance metrics can allow more confidence in the inferred edges than hard thresholding GENIE3’s fully connected network. Figure 6a and b also illustrate the order of magnitude of the number of connections removed by the testing strategy.

After using our empirical testing procedure for edges removal, we stored the number of remaining edges. We then applied hard-thresholding to GENIE3’s ranking in order to create networks containing those same number edges. We observed that the precision of such networks was not as high as with our empirical testing procedure. This reveals that our adjusted *p*-values bring more information than GENIE3’s ranking only, even with a hard-thresholding resulting in the same number of final interactions.

Figure 6b shows computation times required to perform statistical testing on *A. thaliana* dataset, as permitted by DIANE’s online interface. DIANE’s online version is hosted on a Debian 9.13 server with a 256Go RAM, and 2 Intel(R) Xeon(R) Gold 6130 2.10GHz CPUs. The parallel computing for online use allows up to 16 CPU cores (computation time reported in Fig. 6b uses 16 cores).

Altogether, this benchmarking analysis demonstrates an added-value in terms of network precision when edges selection is performed on the basis of *p*-values rather than by hard thresholding, for a limited time of computation.

#### Interactive network analysis and community discovery

The last tab of the application is dedicated to network manipulation and exploration. An interactive view of the network is proposed, showing connections between regulatory genes and their predicted targets. By clicking one of the genes, its inward and outward interactions are shown, as well as its annotation and expression profile across samples.

Network-related statistics are automatically generated, delivering topological insights on genes behaviors and network structure. For instance, in and out degree distribution are displayed, and genes can be ranked based on their number of connections. This ranking might then be used for further identification of hub genes and candidate key regulators in the response of interest. In addition, DIANE extracts gene modules, making use of the Louvain algorithm [62]. The experimenter is then free to visualize the results in the network as color-coded communities, while exploring module-specific expression profiles and GO enrichment analyses. At last, it is possible to download edges and node information as csv dataframes, to be further investigated or opened in popular network visualization tools such as Cytoscape.

**Fig. 7:**
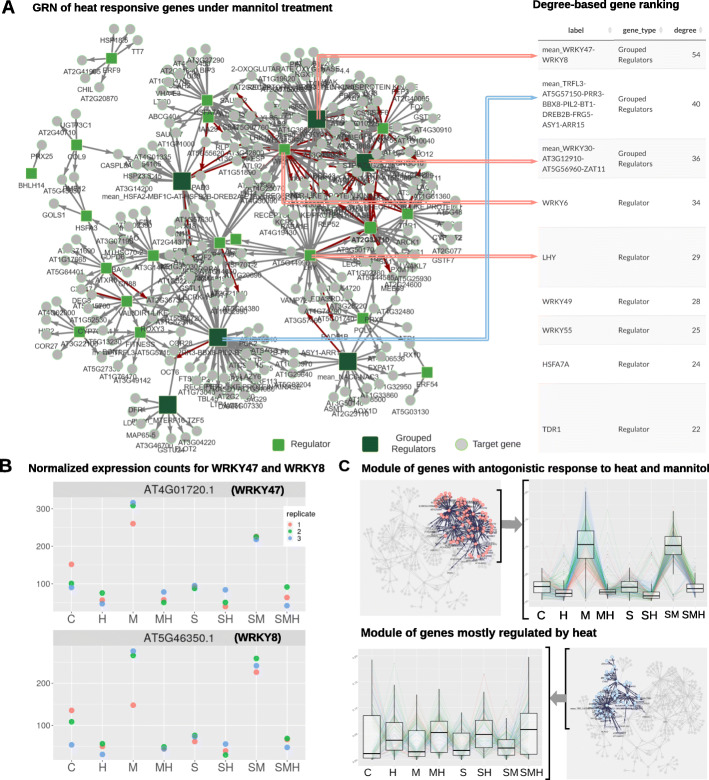
Network inference and exploration with DIANE. **a** GRN on M versus MH DEG using DIANE’s method for GNR inference, and the corresponding degree-based ranking of the nodes. The 11 most connected nodes are presented by order of importance. The regulators mentioned in the network analysis are pointed out by an arrow, the color of the arrow depending on the topological cluster. **b** Expression profiles for WRKY47 and WRKY8, representing the most connected node of the network. **c** Topological modules containing the two most connected groups of regulators are highlighted, juxtaposed to their genes expression profiles

We used the GRN features of DIANE in order to infer a GRN of the response to heat under osmotic stress, environmental conditions that plants are supposed to face more frequently under climate change circumstances. The input list of genes is obtained in DIANE, by calculating DEG between simple osmotic stress and the double heat-osmotic perturbation (M versus HM, FDR <0.01, LFC >2). 640 DEG are detected, among which 363 are up-regulated, 277 are down-regulated, and 45 are transcriptional regulators. Regulators with Spearman correlations over 90% in all available experimental conditions were grouped before network inference, so that a total of 27 regulators are used as predictive variables during inference. For GRN reconstruction, we used Random Forests composed of 4000 trees. A prior network density of 0.03 was defined to select the strongest edges for permutation testing, and edges under a 0.01 FDR were kept in the final network. This network, presented in Fig. 7a, is composed of 289 nodes and 438 edges.

The M versus MH GRN provided by DIANE revealed two interesting groups of regulators, acting as central nodes in their topological modules, and being connected to a large number of target genes.

The most connected regulator of the network is composed by the WRKY47-WRKY8 grouping. Along with other top-ranked WRKY transcription factors (WRKY30, WRKY6, WRKY55), they belong to the topological community of genes that exhibit antagonistic behavior between heat and osmotic stress. The expression values of WRKY8 and WRKY47 in the experiment are presented in Fig. 7b. As already pointed out by our clustering analysis in Fig. 4, those genes undergo a strong induction after mannitol treatment while being repressed by all high temperature conditions. This behavior can also be observed in the intra-module expression profiles in Fig. 7c. Such a module is of high biological interest, as these opposite interactions between drought and high temperature might explain the increased damages observed in the combination of those perturbations [21], and help to understand how heat can suppress the adaptive response of plants to water deficit. Given that WRKY47 and WRKY8 act as a hub in the inferred network, they would be a relevant choice of candidates for experimental pathway validation. Interestingly, WRKY47 has already been identified in rice as a positive regulator of the response to drought [63], strongly reinforcing the validity of the candidate genes from GRN inference in DIANE.

The second most connected node is formed by the regulators TRFL3-AT5G57150-PRR3-BBX8-PIL2-BT1-DREB2B-FRG5-ASY1-ARR15. Those genes, sharing highly correlated profiles across the 24 experimental samples, respond to heat in a clear manner, as well as the other genes inside their community as shown in Fig. 7c. It is worthy to note that PIL2 is a member of a transcription factor family known to be involved in the response to temperature [64] and that DREB2B is a regulator already characterized to act at the interaction between drought and heat stress [65]. The other mentioned regulators offer thus promising leads to be further explored. Three members of the Heat Stress Transcription Factor family (HSFA2 grouped with HSFB2B, and HSFA3) are also found within the genes of the module.

Inside each module, both correlated and anti-correlated expression patterns coexist, which can indicate negative regulation between their gene members. Such opposite variations are captured by the Random Forest algorithm, and allow to go beyond co-expression analysis provided by a clustering approach alone.

### Research reproducibility

For each step of the pipeline, automatically generated reports can be downloaded, rendered on the fly in RMarkdown. They store the users settings, chosen strategies, and display previews of the results. In that way, analysis can be re-run, shared across users, and their settings can be backed-up. The chosen format for those reports is HTML, as it keeps a possibility to interact with data tables, or even manipulate network objects outside of the application. Additional file 2 is an example of report as generated for the network inference described in previous section. Besides, a seed can be set as a global setting of the application, to ensure reproducible runs of the pipeline steps making use of randomness.

### Accessibility

DIANE is a tool designed to be as accessible as possi- ble. However, it can be challenging for users with little programming and command line experience to process raw RNA-Seq data into the expression matrix needed in DIANE. Services such as quality control, read mapping and quantification require to handle large files transfers and intensive computations, which are much less easily set up on online applications. However, local programs such as the Tuxedo suite [66], RMTA [67] or GenePattern [68] represent well documented and adequate solutions to most users in order to produce the expression matrices required in DIANE.

## Conclusions

To summarise this work, we presented an online graphical user interface to easily conduct in-depth analyses on gene expression data from multi-factorial experiments, including gene expression profile clustering and GRN inference. It can be downloaded and installed seamlessly as any R package to run the pipeline locally or from R scripts. Given that all other graphical interface tools found in the literature are (i) more oriented toward co-expression rather than regulation and (ii) do not provide recent advanced methodological frameworks for pathway reconstruction, our application positions itself as a tool of first choice to explore regulatory mechanisms.

The demonstration of DIANE on its companion dataset allowed to better understand the effect of combined heat, osmotic and salinity perturbations on Arabidopsis thaliana, consistently with the original analysis [21]. Similar patterns in gene behaviors were highlighted, such as the predominant influence of heat, and its aggravating effect when combined to dehydration. Moreover, DIANE provided new leads through its network inference features : key genes involved in the response to high temperature under drought were pointed out to be promising candidate regulators for improving crops resistance to arid conditions and climate change.

In terms of computational cost, the final step of DIANE’s pipeline, i.e. the statistical testing of TF-target edges, could be improved. The R implementations of Random forests and permutations in rfPermute are currently being used, but a C++ version could be envisioned to shorten the method’s execution time. Besides, the inference method itself could be subject to improvement in the future. First, combining the results of several inference methods has proven to be as a robust and powerful approach on validated datasets [45,52]. Second, our strategy is particularly well-suited for multi-factorial and perturbation designs, but is not optimal for time series RNA-Seq. Other inference methods specific to time series RNA-Seq data [69] could be available in DIANE, to bring closer to causality in the inferred transcriptionnal interactions. Lastly, it would be valuable to add further functional features in DIANE, notably in order to integrate external information, such as interaction databases, or data from TF binding or chromatin accessibility experiments.

## Availability and requirements

**Project name:** DIANE

**Project home pages:**https://oceanecsn.github.io/DIANEhttps://github.com/OceaneCsn/DIANE

**Operating system(s):** Platform independent

**Programming language:** R

**Other requirements:** Web use : none. Local use: R >4.0.1

**License:** GNU GPL

**Any restrictions to use by non-academics:** none

## Supplementary Information

**Additional file 1** Full description of the procedure of importance measures empirical testing. the files gives more details about the methodological choices for the procedure.

**Additional file 2** Network inference report from the M versus MH GRN. Interactive report generated after network inference and edges testing in DIANE. Slight changes might be observed from the textual description of the network because of the stochasticity inherent to the Louvain, Random Forest, and permutations procedures.

## Data Availability

The RNA-Seq experiment we included to DIANE for demonstration purposes corresponds to the GEO accession GSE146206 and can be found at https://www.ncbi.nlm.nih.gov/geo/query/acc.cgi?acc=GSE146206. The code and benchmark scripts of DIANE are available in the github repositories https://github.com/OceaneCsn/DIANE and https://github.com/OceaneCsn/Benchmarking_DIANE. DIANE largely relies on the CRAN https://cran.r-project.org/ and Bioconductor https://bioconductor.org/ packages repositories. The datasets queried to retrieve validated regulatory interactions are connecTF https://connectf.org/ and RegulonDB http://regulondb.ccg.unam.mx/. The expression data used to infer regulatory networks on *Escherichia coli* were taken from the Many Microbe Microarrays Database at http://m3d.mssm.edu/. Declarations

## Abbreviations

H: High temperature perturbation M: Mannitol perturbation S: Salinity perturbation SM: Salinity and Mannitol perturbations SH: Salinity and High temperature perturbations MH: Mannitol and High temperature perturbations SMH: Salinity, Mannitol and High temperature perturbations DEA: Differential Expression Analysis DEG: Differentially Expressed Genes DIANE: Dashboard for the Inference and Analysis of Networks from Expression data FDR: False Discovery Rate GENIE3: GEne Network Inference with Ensemble of trees GO: Gene Ontology GRN: Gene Regulatory Network LFC: Log Fold Change NGS: Next-Generation Sequencing PCA: Principal Component Analysis RNA-Seq: Sequencing of RNA TF: Transcription Factors TMM: Trimmed Mean of M values WGCNA: WeiGhted Correlation Network Analysis
