# Supplementary material for: Inferring and analyzing gene regulatory networks from multi-factorial expression data: a complete and interactive suite
Source: BMC Genomics. 2021 May 26;22:387. doi: 10.1186/s12864-021-07659-2 (PMC8152307; doi:10.1186/s12864-021-07659-2)
Supplement: Supplementary file 1 — Additional file 1 Full description of the procedure of importance measures empirical testing. the files gives more details about the methodological choices for the procedure. [file 12864_2021_7659_MOESM1_ESM.pdf]

Selecting meaningful importance values from Random Forests inference

Océane Cassan, Sophie Lèbre, Antoine Martin

November 16, 2020

1 Statistical procedure

To assess weather an importance value is significant or not, the `rfPermute` package [1] fits Random Forests and repeatedly shuffles the target gene expression profile so that the null distribution of each regulator influence is estimated. Hence, the empirical p-value of a regulator-gene pair is given by the extremeness of its importance as compared to the estimated null distribution.

As biological networks are known for their pronounced sparsity [2, 3, 4], testing all possible regulator-target pairs would be of very little interest, as well as a waste of computation time. Besides, our preliminary analysis showed that corrections for multiple testing were made unreasonably conservative by the very large number of edges. We therefore propose to create a first graph, topologically consistent with biological network standards, which will be further refined by statistical testing.

More precisely, the steps of the method are :

1. **Inference of the importance values for all regulator-target gene pairs using GENIE3**. The importance metric returned by GENIE3's Random Forests is the total decrease in node impurities from splitting on the variable, averaged over all trees [5]. It requires the target gene expressions to be normalized to a unit variance, so that their regulatory importance measures can be compared without bias. In the GENIE3 framework, it was shown faster and equivalent to another importance metric, the prediction error on the out-of-bag permuted data. Although both can be used for this step, we recommend the use of the second one for consistency reasons regarding the third step.

2. **Selection of the number E of edges based on the inferred regulatory ranking**. The value of E is such as it gives a superior limit to the network density. The total number of possible edges in an oriented regulatory network being $E_{max} = N_{regulators}(N_{genes} - 1)$, and the density being defined as $d = \frac{E}{E_{max}}$, we deduce $E = dN_{regulators}(N_{genes} - 1)$.

Studies such as [4] on state of the art protein-protein interaction structure found that the typical values of density in biological networks lie approximately between 0.1 and 0.001, guiding the user's choice for this parameter.

3. **Empirical p-values are computed for the selected regulatory weights** with the `rfPermute` package. For each gene involved in the selected edges, Random Forests are fitted using its connected regulators as variables, as defined in the network resulting from the first step. The response variable is permuted $nShuffle$ times to build the null distributions. The empirical p-value for an edge is consequently the proportion of the null importance values above the observed importance. We propose a default value $nShuffle = 1000$, but it can be increased for more precise p-value estimations. The importance metric to use in the Random Forests for this step is the prediction error on Out-of-bag examples. Indeed, we observed (data not shown) that, unlike the node impurity measure, prediction error on OOB examples was robust to the reduced number of regulators caused by the selection of E edges only and to over-fitting as well. Moreover, it does not require any expression normalisation, as it is already dealt with within the metric definition.

4. **FDR adjustment** [6] for multiple testing is applied to the set of p-values.

5. Only the **edges above a certain FDR threshold** are kept to be part of the final network.

In brief, the main user-defined parameters are the estimated network density, and the FDR cut-off. Together, they bring much more biological meaning and decision help than an arbitrary importance threshold.

2 Implementation

For the implementation of this method for edges selection, the source code of GENIE3 was modified in order to use the R implementation for Random Forests and allow to change the importance metric. The testing procedure was implemented in a function that benefits from CPU multi-threading to reduce computation time, but it stays the more time consuming step. Graphics that show the p-values distribution and the final number of edges depending on the FDR choice are displayed, providing the user with additional decision guidance.

The method is embedded in DIANE, available either through its user interface, or via functions to run from R scripts, as detailed in the package vignette.

References

[1] Archer, E.: rfPermute: Estimate Permutation p-Values for Random Forest Importance Metrics. (2020). R package version 2.1.81. https://CRAN.R-project.org/package=rfPermute

[2] Koutrouli, M., Karatzas, E., Paez-Espino, D., Pavlopoulos, G.A.: A Guide to Conquer the Biological Network Era Using Graph Theory. Frontiers Media S.A. (2020). doi:10.3389/fbioe.2020.00034

[3] Leclerc, R.D.: Survival of the sparsest: Robust gene networks are parsimonious. Molecular Systems Biology **4** (2008). doi:10.1038/msb.2008.52

[4] Hayes, W., Sun, K., Pržulj, N.: Graphlet-based measures are suitable for biological network comparison. Bioinformatics **29**(4), 483–491 (2013). doi:10.1093/bioinformatics/bts729

[5] Liaw, A., Wiener, M.: Classification and regression by randomforest. R News **2**(3), 18–22 (2002)

[6] Benjamini, Y., Hochberg, Y.: Controlling the false discovery rate: a practical and powerful approach to multiple testing. Journal of the Royal statistical society: series B (Methodological) **57**(1), 289–300 (1995)
