# Supplementary material for: Inferring and analyzing gene regulatory networks from multi-factorial expression data: a complete and interactive suite
Source: BMC Genomics. 2021 May 26;22:387. doi: 10.1186/s12864-021-07659-2 (PMC8152307; doi:10.1186/s12864-021-07659-2)
Supplement: Supplementary file 2 — Additional file 2 Network inference report from the M versus MH GRN. Interactive report generated after network inference and edges testing in DIANE. Slight changes might be observed from the textual description of the network because of the stochasticity inherent to the Louvain, Random Forest, and permutations procedures. [file 12864_2021_7659_MOESM2_ESM.html]

DIANE - Network inference report


Code 

- Show All Code
- Hide All Code

# DIANE - Network inference report

# Dashboard for the Inference and Analysis of Networks from Expression data

---

This report was automatically generated by DIANE to improve research reproducibility.

It contains the main settings and results for the network inference tab of the application.

# Your settings

## Biological question

Normalization method:

```
print(r$norm_method)
```

```
## [1] "none"
```

Input genes for network inference (DEGs from Differential expression tab) :

```
paste(input$input_deg_genes_net, ",", length(r$DEGs[[input$input_deg_genes_net]]) ,"genes")
```

```
## [1] "M MH , 640 genes"
```

Those DEGs were studied via clustering :

```
if(!is.null(r$current_comparison)){
  print(input$input_deg_genes_net == r$current_comparison)
  if (input$input_deg_genes_net == r$current_comparison)
    print(paste(paste(input$input_cluster_genes_net, collapse = ', '), " were the clusters chosen for     inference"))
} else print(FALSE)
```

```
## [1] FALSE
```

Conditions used for the inference :

```
input$input_conditions_net
```

```
## [1] "C"   "S"   "M"   "H"   "SM"  "SH"  "MH"  "SMH"
```

Were genes aggregated to remove splicing awareness? (sum of all transcripts variants for a gene)

```
r$splicing_aware
```

```
## [1] TRUE
```

## Regulators

How many regulators were found among the input genes :

```
if(r$splicing_aware) {
  targets <- get_locus(r$DEGs[[input$input_deg_genes_net]])
}else {
  targets <- r$DEGs[[input$input_deg_genes_net]]
}
regressors = intersect(targets, r$regulators)
length(regressors)
```

```
## [1] 45
```

(When only some clusters are selected, the regulators are still all the regulators present in the input DEGs)

## Grouping correlated regulators

The regulators that were correlated above this threshold (Spearman correlation) were grouped :

```
input$cor_thr/100.0
```

```
## [1] 0.9
```

The grouping was performed using the modules of this correlation network :

```
if(!is.null(r$cor_network)){
    nodes <- r$cor_network$nodes
  nodes$label <- r$gene_info[match(nodes$id, rownames(r$gene_info)), "label"]
  
  visNetwork(nodes, r$cor_network$edges)%>% 
    visNodes(font = list("size" = 35))
}
```

## Inference

Number of genes and regulators after grouping and potential splice variants aggregation:

```
paste("total genes :", ncol(r$networks[[input$input_deg_genes_net]]$mat), "regulators :", nrow(r$networks[[input$input_deg_genes_net]]$mat))
```

```
## [1] "total genes : 615 regulators : 26"
```

Number of trees used for the inference :

```
input$n_trees
```

```
## [1] 4000
```

Importance metric used for the inference :

```
if(input$importance_metric){
  importance = "MSEincrease_oob"
}else
  importance = "node_purity"
importance
```

```
## [1] "MSEincrease_oob"
```

## Fully connected weighted network thresholding

Selected network density and corresponding number of edges :

```
paste("density:",input$density, ",", input$n_edges, "edges")
```

```
## [1] "density: 0.03 , 479 edges"
```

Was statistical testing performed:

```
input$test_edges
```

```
## [1] TRUE
```

# Results

## Network topology

Network view :

```
if(!input$test_edges){
  DIANE::draw_network(nodes = r$networks[[input$input_deg_genes_net]]$nodes,
               edges = r$networks[[input$input_deg_genes_net]]$edges)
}else{
  DIANE::draw_discarded_edges(r$edge_tests$links, 
                       list(nodes = r$networks[[input$input_deg_genes_net]]$nodes,
                            edges = r$networks[[input$input_deg_genes_net]]$edges))
}
```

```
## 479 edges kept in final network
```

Number of nodes and number of edges of the network :

```
graph <- r$networks[[input$input_deg_genes_net]]$graph
paste(length(V(graph)), "nodes,", length(E(graph)), "edges")
```

```
## [1] "289 nodes, 436 edges"
```

Network degrees and betweenness distributions:

```
DIANE::draw_network_degrees(nodes = r$networks[[r$current_network]]$nodes,
                     graph = r$networks[[r$current_network]]$graph)
```

```
## `stat_bin()` using `bins = 30`. Pick better value with `binwidth`.
## `stat_bin()` using `bins = 30`. Pick better value with `binwidth`.
## `stat_bin()` using `bins = 30`. Pick better value with `binwidth`.
## `stat_bin()` using `bins = 30`. Pick better value with `binwidth`.
```

## Highly connected genes

Here are the genes of the network, ranked by degree. The full table, as well as the edges table, can be downloaded directly from the app as a csv file.

```
data <- r$networks[[r$current_network]]$nodes
    
columns <- c("label", "gene_type", "degree", "community")
if (!is.null(r$gene_info)) {
  columns <- unique(c(colnames(r$gene_info), columns))
}
data <- data[order(-data$degree),]
DT::datatable(data[, columns])
```

## Network modules

Number of identified modules :

```
length(unique(r$networks[[r$current_network]]$membership))
```

```
## [1] 10
```

Modules view :

```
nodes <- r$networks[[r$current_network]]$nodes
    
nodes$group <- nodes$community
DIANE::draw_network(nodes = nodes,
             edges = r$networks[[r$current_network]]$edges)
```

Profiles of the modules :

```
if(r$splicing_aware) {
  data <- r$aggregated_normalized_counts
}else{
  data <- r$normalized_counts
}

if(sum(grepl("mean_", 
  r$networks[[r$current_network]]$nodes$id)) > 0){
  data <- r$grouped_normalized_counts
}

DIANE::draw_profiles(data = data,
              membership = r$networks[[r$current_network]]$membership,
              conds = r$networks[[r$current_network]]$conditions)
```

```
## Using gene as id variables
```
